# Cost-benefit analysis of interpersonal therapy and fluoxetine for treating depression and PTSD in primary care settings in Kenya

**DOI:** 10.1186/s12888-026-08239-y

**Published:** 2026-06-12

**Authors:** Easter Olwanda, Daniel Mwai, Muthoni A. Mathai, Rachel L. Burger, Linnet Ongeri, David Bukusi, Anne Mbwayo, Grace Rota, Ammon Otieno, Raymond Rota, Susan M. Meffert, James G. Kahn

**Affiliations:** 1 Futures Health Economics and Metrics, Nairobi, Kenya; 2 University of Nairobi, Nairobi, Kenya; 3 The Executive Office of the President, Nairobi, Kenya; 4 University of California, San Francisco (UCSF), USA; 5 Kenya Medical Research Institute (KEMRI), Nairobi, Kenya

**Keywords:** Depression, PTSD, Interpersonal therapy, Fluoxetine, Benefits: Cost ratio, Kenya

## Abstract

**Background:**

Kenya faces a significant mental health crisis, with 1.9 million reported cases of depression and 10.6% prevalence of post-traumatic stress disorder (PTSD). The economic burden of mental health conditions was 62.2 billion Kenyan shillings in 2021, accounting for 0.6% of GDP. This study performed a cost-benefit analysis (CBA) of Interpersonal Psychotherapy (IPT) and fluoxetine (FLX) for treating depression and PTSD in a primary care setting.

**Methods:**

The SMART-DAPPER project in western Kenya (Kisumu County Referral Hospital) employed a Sequential, Multiple Assignment Randomized Trial design to train non-specialist providers in administering IPT and FLX for adult depression and PTSD. No standard-of-care arm was used. A cost-benefit analysis (CBA) compared intervention costs with income gains from increased productivity, using micro-costing for treatment expenses and the World Bank’s Living Standards Measurement Study to assess productivity. The benefit-cost ratio was calculated over one and ten years with annual relapse rates of 10%, 25%, and 50%. All currency measurements were in KES, converted to USD rate of 118.02 KES per USD.

**Results:**

The study enrolled 1,918 participants: 986 received IPT and 932 received FLX. Remission was achieved after the first round of therapy by 782 (79.3%) and 798 (85.6%), respectively. IPT averaged 11.5 sessions of 60 min, costing in total 5,050 KES (USD42.79); FLX averaged 5.3 sessions of 20 min, costing 2,511 KES (USD 21.28), including the medication. Both treatments increased income, with IPT participants gaining 16,242 KES (137.62 USD) and FLX participants 13,239 KES (112.18 USD) in the first year. Benefit-cost ratios were 3.2:1 for IPT and 5.3:1 for FLX. Over ten years, FLX showed higher CBA ratios (10 to 31:1) than IPT (6 to 18:1).

**Conclusion:**

This study found productivity gains greater than primary care-based treatment costs for IPT and FLX for depression and PTSD in Kenya. FLX demonstrated a more favorable benefit-to-cost ratio. Future research should address the capacity of government health care to sustain delivery of psychological and pharmacological therapy.

**Supplementary Information:**

The online version contains supplementary material available at 10.1186/s12888-026-08239-y.

## Introduction

Kenya has an estimated 1.9 million depression cases, fifth among African countries after South Africa (2.4 million cases), the Democratic Republic of Congo (2.9 million cases), Ethiopia (4.5 million cases), and Nigeria (7.1 million cases) [1]. A recent household survey in western Kenya revealed that 48% of adults had experienced severe trauma, with a post-traumatic stress disorder (PTSD) prevalence rate estimated at 10.6%, defined as six or more on the trauma screening questionnaire (TSQ) [2].

In 2021, mental health conditions imposed a significant financial burden on the Kenyan economy, amounting to 62 billion Kenyan Shillings (KES) (Int$ 572 million), 0.6% of the country’s 2020 gross domestic product. These costs can be broken down into two primary categories: healthcare expenditures (5.5 billion KES) and lost productivity (56.6 billion KES), stemming from premature mortality, absenteeism, and presenteeism (lack of focus at work). Such high costs underscore the urgent need for effective interventions, particularly for common conditions [3].

Public expenditures on mental health are minimal in low and middle-income countries (LMICs) (less than US$ 2 per capita) in 2014 [4]. The World Health Organization (WHO)estimates show that governments allocate less than 1% of their health budget to mental health services [5]. In most LMICs, government spending on mental health is vastly insufficient for the public health burden. In Kenya, less than 6% of the country’s GDP is spent on healthcare, against the 15% agreed in the Abuja Declaration, and mental health comprises just 0.01% of total health spending [6]. The Kenya Mental Health Policy 2015–2030 focuses the following priority actions: increasing the budgetary allocation to mental health services to a minimum of the recommended WHO standards both at national and county health sector budgets and ensuring that the health insurance system does not discriminate against persons with Mental, Neurological and Substance use (MNS) disorders in accessing insurance policies [7]. However, the budget allocation has not been achieved as of 2025.

Delivering mental health services integrated with primary care is recommended by the World Health Organization [8]. Compared to participants who receive traditional non-integrated mental health care, those who receive integrated mental health services have a 20% lower risk of hospitalization, a 15% increase of medication adherence and an 85% higher satisfaction rate [9]. Non-integrative care is a referral-based model where clients in need of mental health services are referred to another clinic or facility for psychiatric care, rather than integrated within the primary care setting. Treatment options like Interpersonal Psychotherapy (IPT) and fluoxetine (FLX) are effective for depression and PTSD, with IPT focusing on improving interpersonal relationships and fluoxetine enhancing serotonin levels [10].

Psychotropic drugs are not consistently available in primary care in most countries. About 20% of countries do not even have the three most commonly prescribed drugs to treat disorders like depression, schizophrenia and epilepsy. Where these drugs are available, high prices are often a barrier to care. Though low-income countries have lower median prices, the difference in prices between low- and high-income countries is only 2 to 5 times while the difference in income is more than ten times, making these drugs relatively less affordable in low income settings [11]. The gap in access to essential psychotropic medicines is huge, posing a challenge to management of mental illnesses. The Kenya Mental Health Policy 2015–2030 states that the Kenya Essential Drug List should include essential psychotropic drugs in adequate quantities and varieties. Specifically, the procurement, storage, and distribution processes should ensure availability of these drugs at all levels of the health system [12].

Economic evaluations are an important tool within the priority setting process, whereby decision-makers allocate resources between existing and/or new healthcare services [13]. Understanding the economic benefits of mental health interventions can lead to optimized strategies for integrating mental health care into primary healthcare systems. The use of a Cost-Benefit Analysis (CBA) framework for policy appraisal is likely to be particularly useful for assessing gains vs. costs, and for allocating resources across sectors, especially when trade-offs need to be made and appraisals across sectors need to be consistent and comparable [13, 14].

By ensuring that mental health services are adequately funded and supported, providers can enhance treatment, accessibility, and quality, contributing to a more resilient healthcare system. In a resource-constrained environment, such informed decision-making is essential for improving both individual and societal well-being. Toward that goal, this study contributes a cost-benefit analysis of IPT and FLX for treating depression and PTSD in primary care settings in Kenya.

## Methods

### Study design

The SMART-DAPPER project in western Kenya was a Sequential, Multiple Assignment Randomized Trial (SMART) that trained and deployed a non-specialist workforce to provide IPT and clinicians (nurses and clinical officers) without an additional mental health specialization, to prescribe FLX for depression and PTSD for adult public sector primary care participants. It leveraged the Depression and Primary-Care Partnership for Effectiveness-Implementation Research Project (DAPPER). Fluoxetine, a selective serotonin reuptake inhibitor (SSRI), is on the WHO list of essential medications for Kenya and is available nationwide to public sector healthcare facilities from Level 4 (Sub-county/District Hospitals), Level 5 (County Referral Hospitals), and Level 6 (National Referral Hospitals), through the Kenya Medical Supplies Authority (KEMSA).

Fluoxetine treatment is associated with decreased symptoms of Major Depressive Disorder (MDD) [15]. Despite the interim development of many other Selective Serotonin Reuptake Inhibitors (SSRIs), none have shown greater clinical efficacy for depression than fluoxetine, and it remains a first-line treatment for depression [16]. Fluoxetine is also a first-line treatment for PTSD [17]. Fluoxetine is on the Kenyan essential drugs list and is commonly used in public sector healthcare.

In the trial, Fluoxetine treatment was initiated and monitored by trained clinical officers and nurses through 8 treatment appointments at baseline, 2 weeks, 4 weeks, and monthly thereafter until month 6. At the first treatment visit, participants were prescribed a daily 20 mg fluoxetine capsule. At each visit, the Patient Health Questionnaire-2 (PHQ-2) was administered to assess the severity of depressed mood and anhedonia over the past 2 weeks. After 1 month, if there were no prohibitive side effects and the PHQ-2 had not improved from the prior visit and was not ‘0’, then the dose was increased from 20 to 40 mg. If not, the participant continued with 20 mg of fluoxetine unless they had prohibitive side effects. At subsequent visits, if they did not have any prohibitive side effects and the PHQ-2 had not improved from the prior visit and was not ‘0’, the dose was increased by 20 mg from the prior visit, up to a maximal dose of 60 mg. 99.4% (*n* = 5126) of delivered fluoxetine doses were 20 mg (the starting dose), 0.4% were 40 mg (*n* = 20) and 0.2% were 60 mg (*n* = 10). If participants missed or delayed treatment visits and subsequently returned to the study, they were dispensed medication as indicated and scheduled for return visits, but the duration of study treatment was not extended, so they were not able to receive all 180 daily doses of medication (6 months).

IPT was selected as the other treatment modality based on published efficacy for depression and PTSD. IPT improves symptoms by addressing problems in social relationships. It is traditionally delivered as weekly one-hour sessions over 12 weeks. IPT has two potential advantages for treating primary care populations in Kenya. First, IPT improves depression and trauma symptoms by addressing problems in social relationships. Given the cultural emphasis on social bonds across Kenya and the region, IPT aligns with local values, making it a scalable treatment. Second, IPT has the advantage of simultaneously treating MDD and PTSD. This is consistent with implementation frameworks that identify the need to consider that fit and potential adaptations of clinical interventions to address the culture and characteristics of communities, organizations, providers, and patients [18]. IPT has been previously adapted and tested in the region and shows strong efficacy [10]. Due to health and safety and travel restrictions created by the COVID-19 pandemic, study participants had the option of receiving some treatment sessions in person or via audio-only on a mobile phone (12).

### Study setting and study participants

The study took place at Kisumu County Referral Hospital (KCRH), a large clinical facility. IPT was delivered by 43 trained IPT providers. Fluoxetine was dispensed by 51 nurses and clinical officers at KCRH. No standard-of-care comparison was used.

Clinical and economic data were collected between September 2020 and July 2022. Participants were recruited from the general outpatient primary care services at the Kisumu County Referral Hospital (KCRH) and other nearby public health facilities. They were included if they screened positive for Major Depression Episode (MDE) and/or PTSD on the Mini-International Neuropsychiatric Interview (MINI), were 18 years of age or older, and could attend weekly IPT sessions/FLX monitoring appointments. The MINI tool has been validated in East Africa [19]. Participants were excluded from the study if they met any of the following criteria:

- **Cognitive Dysfunction**: Individuals with cognitive impairments that hinder their ability to participate in or accurately take FLX, such as a lack of orientation to person, place, time, or situation.
- **Acute Suicidality**: Those with a moderate or high score on the MINI suicidality module, indicating a need for a higher level of care.
- **Substance Use Disorders**: Participants with drug or alcohol use disorders requiring treatment, as indicated by an AUDIT score of 8 or higher or a DAST score of 3 or higher.
- **History of Mania**: Individuals with a history of mania or those requiring treatment for hypomania, as indicated by a positive score on the MINI mania/hypomania module.
- **Pregnancy or Breastfeeding**: Individuals who were pregnant or breastfeeding.
- **Concurrent Mental Health Treatment**: Participants receiving outside mental health treatment during the study phases were excluded. While any mental health treatment was permitted during follow-up phases (which were recorded by the study team), participants were asked not to initiate new mental health treatments during the treatment period. Those who did were classified as dropouts.

Adverse events (AEs) were recorded. There were 764 total AEs. Of these, 43 were considered definitely, probably, possibly or unlikely related to fluoxetine treatment, totaling less than 6% overall: gastrointestinal distress (*n* = 16), sweating (*n* = 5), dizziness (*n* = 3), anxiety/insomnia (*n* = 3), sedation (*n* = 2), sexual dysfunction (*n* = 2), headaches (*n* = 2), fatigue (*n* = 2), skin problems (*n* = 2), tremor (*n* = 1), fever (*n* = 1), seizure (*n* = 1), ob/gyn (*n* = 1), and other (*n* = 2). Two of these events were considered serious, including one episode of gastrointestinal distress (*n* = 1) and one of sexual dysfunction (*n* = 1). No adverse events were reported related to the IPT intervention.

The number of participants diagnosed is presented in Table 1 below.

**Table 1:** Participants diagnosed

	IPT (*n* = 1082)	Fluoxetine (*n* = 1080)	Total (*n* = 2162)
Age, years			
Mean (SD)	36 (11⋅2)	35⋅3 (10⋅8)	35⋅7 (11)
Median (range)	34 (18–85)	33 (18–77)	34 (18–85)
Sex			
Female	977 (90⋅3%)	981 (90⋅8%)	1958 (90⋅6%)
Male	105 (9⋅7%)	99 (9⋅2%)	204 (9⋅4%)
Married or intimate partner	551 (50⋅9%)	568 (52⋅6%)	1119 (51⋅8%)
Formal education			
None	16 (1⋅5%)	19 (1⋅8%)	35 (1⋅6%)
At least some primary	576 (53⋅2%)	541 (50⋅1%)	1117 (51⋅7%)
At least some secondary	389 (36⋅0%)	418 (38⋅7%)	807 (37⋅3%)
At least some higher	101 (9⋅3%)	102 (9⋅4%)	203 (9⋅4%)
Baseline diagnoses (MINI)			
Major depression	1035 (95⋅7%)	1036 (95⋅9%)	2071 (95⋅8%)
PTSD	555 (51⋅3%)	563 (52⋅1%)	1118 (51⋅7%)
Major depression and PTSD	508 (47⋅0%)	519 (48⋅1%)	1027 (47⋅5%)

### Overview of cost-benefit analysis

A CBA is a comparative analysis of all costs to all financial benefits of a program or intervention, all measured in monetary terms [20]. It helps rank and prioritize options based on their costs and benefits to society. Options with a benefit: cost ratio greater than one or benefits exceeding costs are desirable, more so with high ratios or large differences. A CBA can assist in communicating and justifying investment decisions to stakeholders, such as researchers, policy makers, and citizens [21] The benefits: cost ratio is then estimated by dividing the benefits by the costs.

### Data collection

The costing for IPT and FLX treatments in primary care settings was done using micro-costing, which is a cost estimation method employing detailed resource utilization and unit cost data to generate an estimate of economic costs [22]. This process began by collecting data on key resources used for IPT and FLX treatment, including session duration, the number of sessions per treatment course, and treatment providers’ monthly salaries.

For both IPT and FLX, we calculated the total hours per treatment course by multiplying the session duration in minutes by the number of sessions. The core provider costs for IPT and FLX were derived from the Ministry of Health’s monthly salary, including benefits of the treatment providers, adjusted according to the visit time allocated for each therapy type. Additional costs were included in the analysis, such as the cost of screening participants and supervision, which was set at 10% of core provider costs.

Medication costs were also estimated. The number of fluoxetine pills prescribed was recorded, along with the associated cost per pill, which was 5 KES based on government procurement records. The total cost per individual receiving FLX was determined by summing the core provider costs, screening costs, supervision fees, and medication expenses. Finally, for IPT and FLX, we estimated the total cost per individual in Kenyan Shillings (KES) and converted to USD. Detail in the Excel supplement.

For productivity data, the study used a questionnaire adapted from the World Bank **Living Standards Measurement Study (LSMS)** [23]. These results were reported here [24]. LSMS surveys collect data on economic and health dimensions of household well-being, including consumption and income. We examined repeated measures: baseline and follow-up at 3, 6, and 9 months. We calculated the benefits to individuals associated with treatment, both separately for IPT and FLX, and pooled. Specific productivity outcomes were the proportion of participants receiving a monthly income and their mean income among earners. Income was based on wages from work done for a non-household member, agricultural jobs, business, and self-employment in the past month. Productivity gains in the informal and agricultural sectors were converted to monetary values using earnings rates in the local labor market. All currency measurements were in KES, converted to USD rate of 118.02 KES per USD [25]. We derived a monthly mean income measure for each treatment arm by summing the product of percent earning an income and mean income among earners.

### Cost-benefit analysis

For the CBA, we compared intervention implementation costs to changes in productivity. Income comprised earnings from work done for a non-household member, agricultural jobs, business, and self-employment in the past month. Productivity gains in the informal and agricultural sectors were converted to monetary values and included in income. Baseline data characterized the economic status of participants before receiving treatment. At the end of the first line of treatment, the same metrics were assessed to determine changes. The analysis calculated the percentage of participants earning income following treatment, along with the mean monthly income among those earners. The difference between the income metrics before and after treatment was then identified, reflecting the gains attributed to IPT and FLX. Income gains per month were translated to annual and 10-year totals by multiplication and discounting to the present, conservatively ignoring study evidence suggesting rising income among individuals with successful clinical outcomes. The benefit-cost ratio for the first year was the change in income divided by intervention cost. This ratio compared the financial benefits derived from increased income post-treatment with the costs for treatment.

For the ten-year analysis, we assumed that clinical relapse would decrease productivity gains proportionally. We searched for and could not find evidence of these relapse rates in similar settings. Thus, we examined a wide range of potential annual relapse rates: 10%, 25%, and 50%. We adjusted for projected wage growth and inflation and discounted at 3% annually. Detail in Excel supplement.

### Ethics

The study adhered to the ethical principles outlined in the Declaration of Helsinki. The trial was approved by the UCSF Institutional Review Board (IRB), the Kenyatta National Hospital-University of Nairobi Ethics and Research Committee, and the Kenya Pharmacy and Poison’s Board. It was registered on ClinicalTrials. gov (NCT03466346) on 15th March 2018 and conducted in accordance with Human Subjects Protections (HSP) and Good Clinical Practice (GCP). The trial was monitored by the United States National Institutes for Health (NIH) through PPD.

## Results

### Treatment implementation

This study included 1918 participants: 986 in the IPT arm and 932 in the FLX arm. Initial remitters included 782 undergoing IPT and 798 receiving FLX. 104 participants transitioned from IPT to FLX and 100 from IPT to a combination of IPT and FLX. These figures were extracted from visit data for each phase of the treatment. Of those initially receiving FLX, 71 switched to IPT, and 63 to IPT and FLX. In terms of session duration, IPT typically involves longer sessions (60 min) compared to FLX (20 min). Participants undergoing IPT had a mean of 11.5 sessions per treatment course; those on FLX had 5.3 sessions. This resulted in higher total treatment time for IPT (11.5 h) compared to FLX (1.8 h).

Clinical results were recently published. In brief, at treatment end, the relative risk for major depression was 0.15 for IPT and 0.01 for FLX, and for PTSD 0.20 and 0.14, respectively. FLX efficacy was statistically significantly superior to IPT [26].

### Cost results

The study compared the costs associated with IPT and FLX. A total of 986 participants received IPT, while 932 received FLX. The total cost per individual for IPT was 5,050 KES ($42.79 at prevailing currency exchange rates) compared to 2,511 KES ($21.28) for FLX. (Table 2). The core provider costs per full treatment varied, with IPT at 3,915 KES compared to 1,060 KES for FLX. Additional costs, such as screening and supervision, were accounted for. Medication costs were substantial for FLX, with an average of 140 pills prescribed, costing 700 KES per individual.

**Table 2:** Cost of IPT and FLX treatment, primary care, Kisumu, Kenya

	Overall cost by Arm	
	IPT	FLX
**First Line Therapy (Remitters)**	**(** ***N*** ** = 782)**	**(** ***N*** ** = 798)**
Session duration (minutes)	60	20
Sessions per treatment course	11.5	5.3
Hours per treatment course	**11.5**	**1.8**
Monthly salary for treatment provider (KES)	51,070	90,000
Core provider costs per full treatment (KES)	3915	1060
Cost of screening (KES)	150	150
Supervision at 10% (KES)	392	106
Medication # pills (20 mg)	-	140
Medication cost (5 KES)	-	700
Cost per individual (KES)	4,457	2,066
Full Cohort*	*N* = 986	*N* = 932
Cost per individual (KES)	5,050	2,511
Cost per individual (USD)	$42.79	$21.28

* The number of fluoxetine pills and medication costs are reported per participant

* Full details of costs for individuals requiring further treatment are provided in the Excel supplement

### Productivity results

Both IPT and FLX participants reported increased income. Accounting for both the likelihood of earning an income and the amount of that income, individuals treated with IPT gained KES 16,242, and FLX gained KES 13,239 in year 1. IPT remitters reported larger income gains and greater reductions in absenteeism and presenteeism than did non-remitters. More details on the productivity benefits have been published here [24, 26].

### Cost-benefit analysis

Overall, both treatments provided net economic benefits, with FLX demonstrating a higher benefit-cost ratio than IPT (Table 3). During the first year, for IPT, the percentage of participants earning income increased from 54.9% to 59.8%, with an annual income among earners rising from KES 50,280 to KES 73,320, resulting in a per-participant income gain of KES 16,242 against a treatment cost of KES 5,050, yielding a benefit-to-cost ratio of 3.2:1. FLX showed a percentage increase from 0.545 to 0.615, with annual earnings growing from KES 46,800 to KES 63,000, leading to a per-patient income gain of KES 13,239 versus a treatment cost of KES 2,511, resulting in a benefit-to-cost ratio of 5.3:1.

**Table 3:** Cost-benefit analysis for IPT and FLX (Year 1), treatment of depression & PTSD, primary care, Kisumu, Kenya

	Before Treatment			End of First Line Treatment			Difference (Gain)	Treatment cost	Benefit: Cost Ratio
Initial Therapy	% earning income (prior month)	Annual income among earners (mean, KES)	Annual income among all participants (mean, KES)	% earning income (prior month)	Annual income among earners (mean, KES)	Annual income among all participants (mean, KES)	Annual income among all participants (mean. KES)	Across all treatment paths (KES)	
IPT	54.9%	50,280	27,604	59.8%	73,320	43,845	16,242	5,050	3.2
FLX	54.5%	46,800	25,506	61.5%	63,000	38,745	13,239	2,511	5.3

### Sensitivity analysis

The sensitivity analysis evaluated the benefit-cost ratios of IPT and FLX treatments for depression and PTSD over ten years, varying annual relapse rates (Table 4). For IPT, the income gains adjusted for inflation were KES 91,608 with a benefit: cost ratio of 18.1 at a 10% relapse rate, KES 55,215 with a benefit: cost ratio of 10.9 at 25%, and KES 30,539 with a benefit: cost ratio of 6.1 at 50%. In contrast, FLX showed income gains of KES 76,966 and a benefit: cost ratio of 30.7 at a 10% relapse, KES 45,908 with a benefit: cost ratio of 18.3 at 25%, and KES 25,051 with a benefit: cost ratio of 9.98 at 50%. Thus, FLX had higher benefit: cost ratios across all relapse rates. Details are available in an Excel supplement.

**Table 4:** Cost-benefit analysis for IPT and FLX (Ten years, varied relapse rates), treatment of depression & PTSD, primary care, Kisumu, Kenya

	Annual Relapse 10%		Annual Relapse 25%		Annual Relapse 50%	
	Income Gain (KES) *	Benefit: Cost Ratio	Income Gain (KES)	Benefit: Cost Ratio	Income Gain (KES)	Benefit: Cost Ratio
IPT	91,608	18.14	55,215	10.93	30,539	6.05
FLX	76,966	30.65	45,908	18.28	25,051	9.98

## Discussion

We compared delivery costs to increases in economic productivity for two primary care-based treatments for depression and PTSD in Kenya, finding that economic gains exceed implementation costs by ratios of 3.2:1 for IPT and 5.3:1 for FLX in the first year. These benefit-cost ratios are projected to increase to as high as 30.05 over ten years, depending on the therapy and assumptions about relapse rates. Thus, the potential benefits of mental health interventions like IPT and FLX for treating depression and PTSD are evident, of notable importance in resource-limited settings such as Kisumu, Kenya. Both treatments offer impressive clinical outcomes, making them valuable options for improving mental health care. Furthermore, the economic gains reported by both treatment groups highlight the potential for mental health interventions to contribute to increased productivity in the workforce. This has broader implications for economic development, particularly in low-resource settings where mental health issues are often stigmatized and underfunded. By demonstrating the tangible financial benefits of effective treatment, this study could catalyze increased investment in mental health services.

For mental health care in Kenya more generally, benefit-to-cost ratios vary due to the diverse components involved in implementation, which present important considerations for policymakers and healthcare providers. The Kenya Mental Health Investment case shows that while investing in depression interventions generates economic benefits, the benefits-to-cost ratio is relatively modest at 2.2:1 at 20 years and 1.8:1 at 10 years. This difference from our results can be attributed to several factors, including the costs associated with inpatient care for individuals with severe mental health conditions, the frequent need for outpatient visits for effective management, the ongoing expenses of essential psychotropic medications, and the broader program costs encompassing management, administration, and training of healthcare providers [27]. In addition, our trial focused on comparing real-world treatment strategies suitable for non-specialist primary care, with no untreated controls. Thus, we could not adjust for spontaneous remission, which would likely have decreased the productivity gains associated with treatment.

While the fluoxetine arm showed a smaller change in annual income compared to the IPT arm, its substantially lower delivery cost resulted in a higher benefit-to-cost ratio. Consequently, the economic advantage observed for fluoxetine is driven primarily by its lower implementation cost rather than by superior productivity gains. Conversely, the productivity improvements associated with IPT were slightly higher, but the higher implementation (mainly labor) cost of IPT delivery reduced its relative economic return.

In our analysis, starting treatment with FLX demonstrated a more favorable benefit-to-cost ratio than for IPT, especially in the sensitivity analysis, which indicates that it may be more sustainable in the long term. This raises a pivotal question about balancing cost-effectiveness with treatment intensity [18, 19]. Our sensitivity analysis, which revealed that initial FLX consistently demonstrated higher benefit-to-cost ratios compared to initial IPT across different relapse rates, supports this perspective. This finding suggests that while IPT may offer in-depth treatment, FLX is a more sustainable option for scaling interventions within the Kenyan healthcare system.

Both IPT and Fluoxetine enhance equity. IPT is valuable for individuals with trauma histories or gender-based violence exposure, groups often underserved in health systems. Studies of task-shared mental health services in low- and middle-income countries show that psychosocial interventions delivered by non-specialists can achieve strong clinical outcomes, particularly for depression and trauma-related disorders [28]. Conversely, fluoxetine may enhance equity by improving access in rural or low-staffed facilities where specialist providers are scarce. This aligns with global evidence that medication-based treatments, when appropriately monitored, can be efficiently scaled within primary care platforms [29].

Relapse prevention emerges as another major determinant of long-term economic outcomes. Global ROI analyses indicate that sustaining treatment effects over time generates far larger economic returns than initial short-term remission alone [29]. Similarly, evidence from the SMART-DAPPER trial shows high remission maintenance six months after treatment end for both IPT and fluoxetine, particularly among those who remitted initially [28]. This reinforces the importance of integrating mental health care within chronic disease platforms such as HIV or NCD programs to support long-term recovery in settings where follow-up otherwise falters.

Finally, presenting mental health care as a driver of economic development may help reposition it within national policy priorities. Global economic analyses indicate that each dollar invested in treating depression and anxiety can yield returns of 3:1 to 5:1 when productivity gains and improved health outcomes are considered [29]. Kenya’s emerging mental health reforms and the government’s interest in scaling depression treatment create an opportunity to integrate IPT and fluoxetine into broader development and productivity frameworks. Aligning costs with existing health sector financing mechanisms such as community health programs, maternal and child health services, or HIV platforms, could improve long-term sustainability and ensure interventions reach populations most in need.

This study has several limitations to consider when interpreting the findings. First, the economic model relies on assumptions that may not fully capture the complexity of mental health treatment. In particular, it does not account for heterogeneity in treatment response. Individuals who respond less well to IPT or fluoxetine may require prolonged care, additional services, or alternative interventions, leading to higher health system and out-of-pocket costs than those reflected in the model. As a result, the estimated cost–benefit ratios may be optimistic for subgroups with poorer clinical outcomes.

A further limitation is the failure to explicitly consider the government’s goals for investing in mental health. While the cost–benefit analysis focused on economic returns from income gains following treatment, it did not capture the intrinsic value individuals place on improvements in mental health and well-being. Non-market benefits—such as improved quality of life, reduced stigma, and enhanced social functioning—were therefore not reflected in the analysis.

A related limitation is the lack of explicit willingness-to-pay (WTP) estimates from both patients and the health care system. By focusing on income gains following treatment, the analysis does not capture the value individuals place on non-market benefits, such as improved well-being and quality of life. This omission underestimates the true societal value of mental health interventions.

Finally, initial training costs for IPT and fluoxetine-related care, including provider training through structured modules on clinical protocols, patient management, and ethics, as well as supervision through mentoring, case reviews, and quality assurance, were excluded from the analysis, potentially leading to an underestimation of implementation costs, particularly during early rollout.

## Conclusion

In conclusion, while both IPT and FLX have their merits, and importantly both work clinically and economically, the choice between them may consider economic viability and patient preference. Future studies should explore long-term outcomes and the implications of treatment transitions, as well as the integration of mental health services into primary care, to enhance accessibility and effectiveness in diverse populations.

## Supplementary Information

Below is the link to the electronic supplementary material.

Supplementary Material 1

## Data Availability

The anonymized data used in this study are not publicly available due to ethical reasons but are available with corresponding author upon reasonable request.

## Abbreviations

CBA: Cost-Benefit Analysis
FLX: Fluoxetine
GCP: Good Clinical Practice
GDP: Gross Domestic Product
HSP: Human Subjects Protections
IPT: Interpersonal Psychotherapy
IRB: Institutional Review Board
KEMRI: Kenya Medical Research Institute
KEMSA: Kenya Medical Supplies Authority
KES: Kenya Shillings
LMICs: Low and Middle-Income Countries
LSMS: Living Standards Measurement Study
MDD: Major Depressive Disorder
MDE: Major Depression Episode
MINI: Mini-International Neuropsychiatric Interview
MNS: Mental, Neurological and Substance use
NIH: National Institutes for Health
PTSD: Post-Traumatic Stress Disorder
SMART: Sequential, Multiple Assignment Randomized Trial
SSRI: Selective Serotonin Reuptake Inhibitor
TSQ: Trauma Screening Questionnaire
UCSF: University of California, San Francisco
USD: United States Dollar
WHO: World Health Organization
